# Are Gender Differences in Upper-Body Power Generated by Elite Cross-Country Skiers Augmented by Increasing the Intensity of Exercise?

**DOI:** 10.1371/journal.pone.0127509

**Published:** 2015-05-22

**Authors:** Ann Magdalen Hegge, Kenneth Myhre, Boye Welde, Hans-Christer Holmberg, Øyvind Sandbakk

**Affiliations:** 1 Center for Elite Sports Research, Department of Neuroscience, Norwegian University of Science and Technology, Trondheim, Norway; 2 Department of Sports and Physical Education, North-Troendelag University College, Levanger, Norway; 3 Swedish Winter Sports Research Centre, Mid Sweden University, Östersund, Sweden; University of Rome Foro Italico, ITALY

## Abstract

In the current study, we evaluated the impact of exercise intensity on gender differences in upper-body poling among cross-country skiers, as well as the associated differences in aerobic capacity, maximal strength, body composition, technique and extent of training. Eight male and eight female elite skiers, gender-matched for level of performance by FIS points, carried out a 4-min submaximal, and a 3-min and 30-sec maximal all-out test of isolated upper-body double poling on a Concept2 ski ergometer. Maximal upper-body power and strength (1RM) were determined with a pull-down exercise. In addition, body composition was assessed with a DXA scan and training during the previous six months quantified from diaries. Relative to the corresponding female values (defined as 100%), the power output produced by the men was 88%, 95% and 108% higher during the submaximal, 3-min and 30-sec tests, respectively, and peak power in the pull-down strength exercise was 118% higher (all *P*<0.001). During the ergometer tests the work performed per cycle by the men was 97%, 102% and 91% greater, respectively, and the men elevated their cycle rate to a greater extent at higher intensities (both *P*<0.01). Furthermore, men had a 61% higher VO_2_peak, 58% higher 1RM, relatively larger upper-body mass (61% vs 56%) and reported considerably more upper-body strength and endurance training (all *P*<0.05). In conclusion, gender differences in upper-body power among cross-country skiers augmented as the intensity of exercise increased. The gender differences observed here are greater than those reported previously for both lower- and whole-body sports and coincided with greater peak aerobic capacity and maximal upper-body strength, relatively more muscle mass in the upper-body, and more extensive training of upper-body strength and endurance among the male skiers.

## Introduction

The endurance sport of cross-country skiing involves the whole body, with the workload shared between the upper and lower extremities in a manner that varies with terrain, skiing speed, technique utilized and capacity of the skier. Recently, gender differences in cross-country skiing performance have been reported to become more pronounced as the contribution of poling increases [1], indicating that these differences may primarily be caused by the upper body. With the double-poling technique, where all propulsion is generated through the poles, gender differences in absolute power were as high as 67%, compared to 62% and 58% with the G3 skating and diagonal stride techniques, respectively, where the contribution of the legs is greater, and only 54% during running, where all propulsion is generated by the legs [1].

This gender difference during running is comparable to the gender differences observed in other endurance sports that rely on leg propulsion [2–4]. Furthermore, these gender differences appear to be independent of exercise duration [4], which is also the case for roller ski skating with the G3 technique, where the workload is shared relatively equally between the upper and lower extremities [5]. Whether such gender differences are independent of the duration and intensity during upper-body exercise as well remains to be examined. However, since previous research revealed that gender differences in strength and power are greater for the upper than lower extremities [6, 7], it can be expected that increasing intensity further accentuates these differences with exercise that solely involves the upper body.

Previously, a large proportion of the gender difference in endurance performance has been attributed to the greater aerobic capacity of men, resulting from their higher levels of hemoglobin and more extensive muscle mass [8, 9]. Indeed, gender differences in endurance performance are reduced by normalization for fat-free or lean mass, which often actually obliterates these differences in sprint performance [10, 11]. Although the impact of such normalization on leg work is clear, findings on its impact with respect to upper-body power and strength performance are less consistent [6, 12–14].

In one report on recreationally active men and women, the men were found to have relatively more muscle mass in their upper body [15]. If this is also the case for athletes with a well-trained upper body, gender differences in the performance of upper-body exercise may be expected to be greater than those associated with leg exercise. In addition to such biological differences, there may also be gender differences with regards to training, especially in the case of cross-country skiing. Daily training involves varying terrain, as well as different modes of exercise (e.g., skiing, running, cycling) and skiing techniques (classical and skating, with all their sub-techniques). Consequently, differences in the distribution of upper- and lower-body loading during training may augment the physiological differences, as well as differences in skiing technique between men and women.

The primary aim of the current study was to test the hypotheses that gender differences in power production by cross-country skiers performing isolated upper-body poling are significantly enhanced by elevating the intensity, that these differences are greater than those previously reported in connection with lower- and whole-body exercise, and that they coincide with corresponding differences in the upper-body aerobic capacity, strength and muscle mass. For this purpose we compared power production, peak aerobic capacity, maximal strength, body composition, technique and the extent of training between elite male and female cross-country skiers.

## Methods

### Subjects

The eight male and eight female elite Norwegian cross-country skiers, who participated in this study, all competed at the national and international level and men and women were matched for performance on the basis of their average International Ski Federation’s (FIS) ranking points [1, 5]. Their characteristics are documented in Table 1.

**Table 1:** Anthropometric, physiological, training and performance characteristics (means ± SD) of the 8 male and 8 female elite cross-country skiers who participated in the study.

	Men	Women
Age (yrs)	20.4 ± 2.2	22.7 ± 3.2
Body height (cm)	183 ± 4*	170 ± 6
Body mass (kg)	78.4 ± 7.3*	65.2 ± 5.9
VO_2_max (mL min^-1^ kg^-1^)	73.9 ± 5.3*	62.0 ± 1.5
Training hours per year	614 ± 93	598 ± 93
FIS points	128 ± 48	131 ± 36

*FIS* International Ski Federation

* Significantly different from the corresponding value for women

### Ethics statement

The experimental procedures were approved by the Regional Ethics Committee for Medical and Health Research in Trondheim, and the protocol and procedures were verbally explained to each subject prior to obtaining written consent to participate.

### Experimental design

All skiers completed a 4-min submaximal test, a 3-min maximal performance test and a 30-s all-out test employing isolated upper-body poling on a ski ergometer, all in a single session. Power output, kinematics and cardiorespiratory variables were monitored continuously, and the blood lactate concentration was assessed after each test. On another day, peak power and maximal strength (1RM) were tested utilizing a pull-down exercise designed to imitate the double-poling movement. In addition, body composition (as determined by a DXA scan) and information on training during the six months prior to testing (May-October) were collected from training diaries. All tests were performed within a four-week period before the start of the cross-country skiing season, and all skiers were familiar with the ski ergometer from use in their daily training.

### Instruments and materials

**Fig 1:**
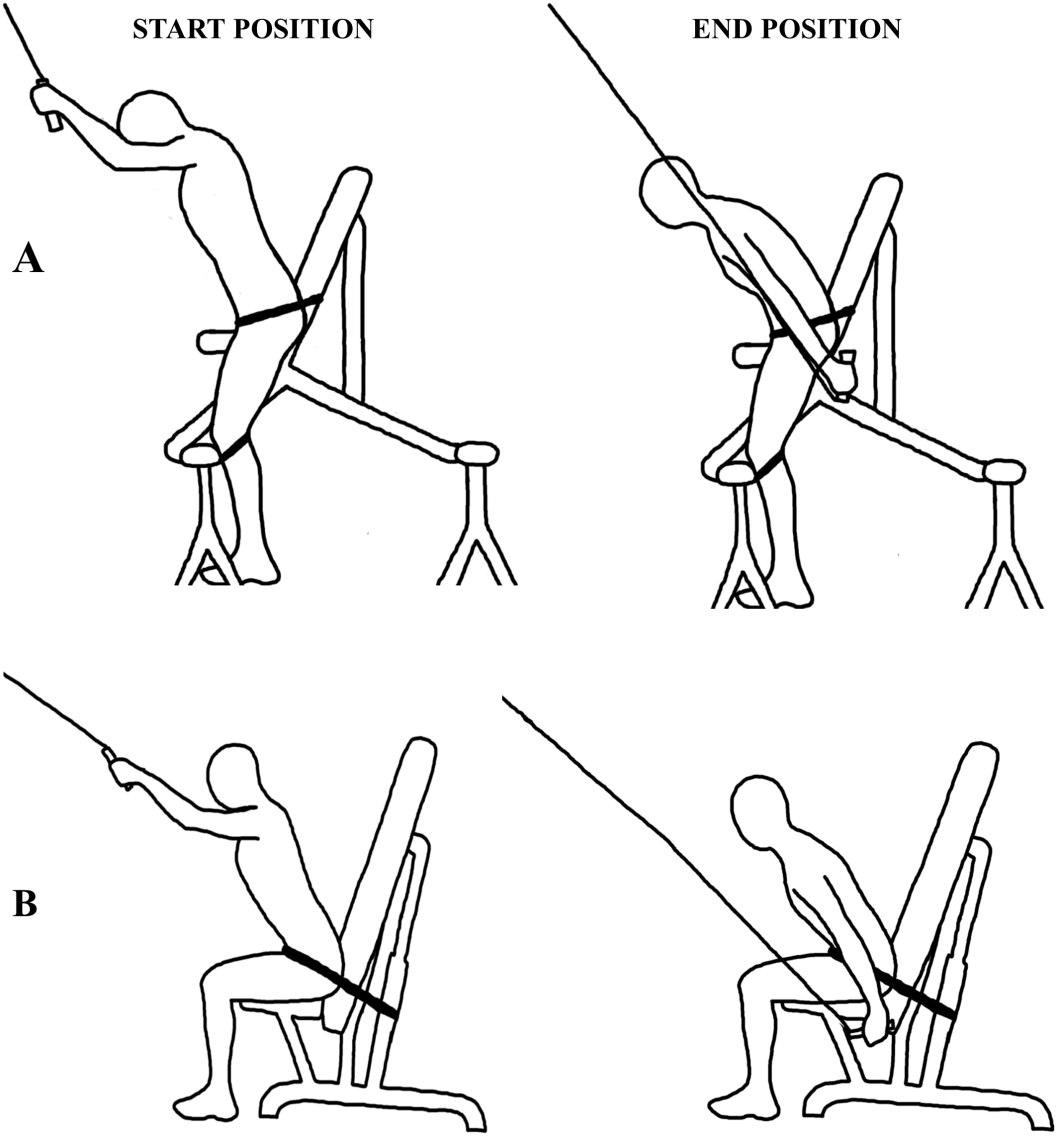
Illustration of the set-ups for upper-body poling (A) and pull-down strength (B).

Upper-body poling was performed on a modified Concept2 SkiErg apparatus (Morrisville, VT, USA), with the flywheel set at the lowest possible drag. The skiers sat on an elevated bench, in front of the SkiErg in order to isolate the lower body from the double-poling movement (see Fig 1A). The distance between the bench and ergometer and elevation of the bench were adjusted to the height of each individual skier, so that the angles at the elbow, shoulder, hip and knee in the starting position were the same for all participants. The seat of the bench was horizontal and the back rest at a 120° angle to the seat. The skier’s pelvis was strapped to the back rest, allowing free motion of the entire upper body. Additional straps around the knees locked the ankle and knee joints, thereby minimizing the contribution of the legs.

Activation of the m. *vastus lateralis*, m. *biceps femoris* and m. *gastrocnemius lateralis*, measured by surface electromyography (Noraxon USA Inc., Scottsdale, AZ, USA) according to SENIAM guidelines [16], was shown to be negligible. Power output and cycle rate were monitored by the ergometer’s internal software and validated using a force cell (Noraxon USA Inc., Scottsdale, AZ, USA) situated on the rope of the pulling device and a motion capture system consisting of infra-red Oqus cameras (Qualisys AB, Gothenburg, Sweden).

Pull-down strength (Fig 1B) was tested on an adjustable bench placed in front of a pull-down apparatus with the same handles as on the Concept2 SkiErg. The back rest was at a ~120° angle with the seat. The skier sat on the bench with a ~90° angle at the knees and was strapped around the hips to isolate the upper body. In this way, the movement in the strength test was similar to that of poling. The friction in the cable apparatus, as measured with the Noraxon force cell, did not change with increasing weight. Peak power in Watts was assessed with a linear encoder and analyzed with the corresponding acquisition software (MuscleLab, Ergotest Technology AS, Langesund, Norway).

Respiratory variables were assessed employing open-circuit indirect calorimetry, with expired gas passing through the mixing chamber being monitored continuously (Oxycon Pro apparatus, Jaeger GmbH, Hoechberg, Germany). This instrument was calibrated against both ambient air and a commercial mixture of O_2_ (16.00%) and CO_2_ (5.85%) at the start of each test day. The O_2_ and CO_2_ contents of the ambient air were recorded and the flow transducer was calibrated using a 3-L high-precision calibration syringe (Calibration syringe D, SensorMedics, Yorba Linda, CA, USA). Heart rate (HR) was monitored continuously with a Suunto t6c heart rate monitor (Suunto Oy, Vantaa, Finland) and synchronized with the VO_2_ measuring system. Blood samples (20 μL) from the fingertip were analyzed using the Biosen C-Line Sport lactate assay system (EKF-diagnostic GmbH, Magdeburg, Germany).

Movements of the upper body were monitored in three dimensions by the Qualisys motion capture system. Seven Oqus cameras recorded the positions of 12 passive reflectors on the upper body and ski ergometer at a rate of 100 frames per second and for a total of 30 seconds. These reflectors were positioned on the joints at the wrist (styloid process of the ulna), elbow (lateral epicondyle of the humerus), shoulder (lateral end of the acromion process) and hip (the greater trochanter) on the left side of the body only, as well as at the fixed origin of the pulling ropes at the top of the ergometer and just above the handles at the end of these ropes. Since the poling movement was regarded as symmetrical, the markers were placed only on one side of the body.

These markers provided coordinates in the X (mediolateral), Y (anteroposterior), and Z (vertical) planes, but for simplicity, only flexion-extension movements were taken into consideration, i.e., movement in the X direction was disregarded. Angular velocity was calculated by differentiation of the positional data using a 5-point filter. The kinematic data were low-pass filtered with a cut-off frequency of 25 Hz (8th-order, zero-lag Butterworth). The data were collected in acquisition software (Qualisys Track Manager) and then evaluated in a MATLAB (R2013a) program designed specifically for the analysis of the double-poling movement on the Concept2 ski ergometer.

### Test protocols

A 10-min run on a treadmill and a 10-min movement-specific low-intensity warm-up (both at <75% of HRmax) were performed on the ski ergometer. Thereafter, each skier completed a 4-min submaximal stage of double poling at a low intensity (~75% of HRmax and 13 on Borg’s RPE scale [17]), a 30-s all-out and a 3-min maximal performance test, with a 5- and 10-min break, respectively. During these breaks, the skiers were allowed to walk or jog at very low intensity to enhance blood circulation and thereby promote removal of lactate. Before both the 30-s and the 3-min test, the athlete double-poled for 1–2 minutes on the ski ergometer, including a 10-s sprint to activate the fast-twitch muscle fibers. The skiers were instructed to perform the 3-min test at an even maximal pace until reaching exhaustion and the 30-s test with maximal effort from the very beginning. During all tests, they could see their power output continuously on the ergometer screen.

The average power outputs during these latter two tests were taken as indicators of test performance. The cardiorespiratory variables were averaged over the final minute of the submaximal stage and the two highest consecutive 10-s VO_2_-values during the 3-min test were defined as VO_2_peak. A 3-min performance test on a ski ergometer has recently been reported to provide a reliable measure of peak aerobic capacity [18]. Blood lactate was determined immediately after the submaximal stage and approximately 1.5 minutes after both the 30-s and 3-min test. On a separate day, an incremental running test to exhaustion was performed in accordance with standard procedures to indicate VO_2_max and HRmax [19].

Prior to the strength test, each skier warmed up by running on a treadmill for 10-min at a low intensity (<75% of HRmax). Warm-up for the pull-down movement was performed at gradually increasing loads: two sets of two repetitions at 40%, five repetitions at 60%, and three repetitions at 80% of the estimated 1RM. Thereafter, the load was further increased by 1.25–2.5 kg per lift until the skier failed to execute the exercise correctly. Between sets, recovery was allowed for two minutes. Finally, three repetitions each at 40, 60 and 80% 1RM, with two minutes of rest in between, were completed with the best trial subjected to further analysis. At the command of the test supervisor, the subject pulled as forcefully as possible. The pull was considered acceptable if the handle bar passed the hip joint. Peak power was calculated as the product of force and average velocity during the lift.

### Kinematics

The work per cycle was calculated as the power output divided by the cycle rate as determined by the internal software of the validated ski ergometer. The poling phase was defined as the phase from the start to the end of the poling phase, i.e., from the minimal to the maximal distance between the markers on the handles and the fixed markers on top of the ergometer. In all cases, measurements were performed for 30 seconds and average values obtained for at least 15 cycles involving steady production of power.

Elbow and shoulder angles were defined as the angle between the two segments on either side of the respective joint. Trunk angle, defined as the angle between the trunk segment and the horizontal plane at the hip level, was calculated from the positions of the shoulder and hip markers. The difference between the angles at the start and end of stroke defined the range of motion of each segment during the poling phase. The angular velocities were calculated as range of motion per unit of time.

### Body composition

Body mass and composition were assessed by dual-energy X-ray absorptiometry (Encore 2007, Version 11.4, General Electric Medical Systems, Madison, WI, USA). Total, lean and fat mass and bone mineral content were determined for the legs, trunk, arms and head and are presented here for the upper body (arms and trunk) and the whole body. In accordance with the manufacturer’s guidelines, the equipment was calibrated using a phantom prior to each measurement. The validity of the procedure was confirmed by comparing the DXA values with manual measurements of body height (Harpenden Portable Stadiometer, Holtain Ltd., Crosswell, UK) and body mass (Tanita BC-545, Tanita Corp Inc., Tokyo, Japan), which resulted in intraclass correlations coefficients of 0.99 and 0.99, respectively.

### Training data

Individual training from May until October was quantified on the basis of the skiers’ personal training diaries, according to the session goal method [20, 21], regarded as providing a valid and accurate measurement of the duration and intensity of cross-country skiers’ training [22]. Training was categorized as follows: low-intensity (heart rate < 81% of HRmax), moderate-intensity 81–87% of HRmax), high-intensity (>87% of HRmax), as well as speed and strength training. Training modes were categorized as roller skiing with the classical or skating technique, running, or other (e.g., cycling, kayaking). Additional information concerning strength and roller ski training was acquired using a questionnaire and qualitative interviews with the individual athletes, where each described the following aspects of training in greater detail: relative amounts of strength training for the arms, core and legs (totaling 100%); ratio of maximal (1–10 repetitions) versus submaximal (11–30 repetitions) strength training; ranking of the extent to which the major sub-techniques of classic and skating roller skiing were utilized (1 for most, 3 for least); as well as the extent to which double poling was the primary technique employed during the sessions of classical roller skiing. These assessments, designed to provide additional insight into possible differences in training by the men and women, were interpreted qualitatively, since the questionnaires employed have not been validated.

### Statistical analyses

All sets of data were found to be normally distributed and means ± standard deviations are presented. A two-way mixed factorial ANOVA, with repeated measures of intensity and gender as the independent variables, was applied to determine the impact of intensity and to analyze possible interaction effects between gender and intensity. In case of a significant interaction effect, gender differences were tested using an independent samples *t*-test. Mauchly’s test of sphericity and Levene’s test of equality of error variances were applied to control for equal and unequal variances. In all cases, the values for the women were set at 100%. Statistical significance was set at an alpha level of 0.05. All statistical analyses were processed using IBM SPSS Statistics for Windows, Version 21.0 Software (IBM Corp., Armonk, NY, USA).

## Results

### Power output and maximal strength

**Fig 2:**
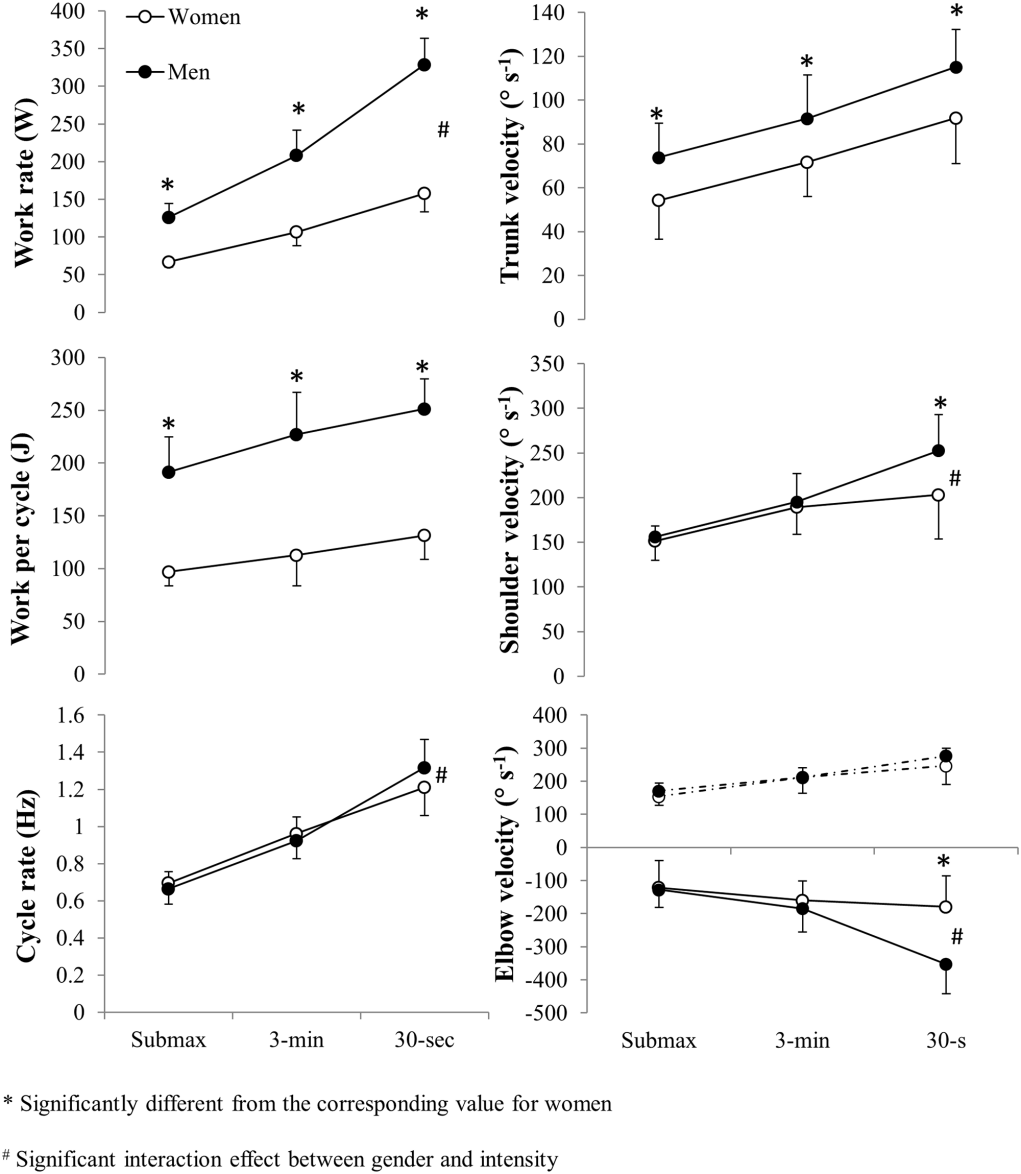
Power output, work per cycle, cycle rate and velocities of trunk flexion, shoulder extension and elbow flexion (negative value) and elbow extension (positive value) for the 8 male and 8 female cross-country skiers during upper-body poling at submaximal intensity (submax), and during a 3-min maximal performance test (3-min) and a 30-sec all-out test (30-sec).

Power output produced by the men was 88%, 95% and 108% higher during the submaximal, 3-min and 30-sec tests, respectively (all *P* < 0.001, Fig 2). In connection with the maximal strength test, the men exhibited a 58% higher 1RM (85.0±9.6 vs 53.7±6.2 kg, *P* < 0.001), whereas their peak power during this test (reached at 80% 1RM by both genders) was 118% higher (1157±176 vs 530±63 W, *P* < 0.001). The gender differences in power output were reduced to 44%, 49%, 61% and 65% when normalized for total upper-body mass (see body composition values at a later stage), and to 33%, 37%, 48% and 54% when normalized for lean upper-body mass (all *P* < 0.001, Table 2). These differences were enhanced significantly with increasing intensity and the peak power produced in the pull-down strength test was significantly greater than that produced during all three ergometer tests (all *P* < 0.001).

**Table 2:** Relative power output and kinematics (means ± SD) for the 8 male and 8 female elite cross-country skiers during upper-body poling at submaximal intensity and during a 3-min maximal performance and 30-sec all-out test.

		Intensity		
		Submaximal	3-min	30-s
Power output (W)	Men	126 ± 19*	208 ± 34*	329 ± 35*
	Women	67 ± 7	107 ± 18	158 ± 24
Power output (W kg^-1^)^TUM^	Men	2.6 ± 0.2*	4.4 ± 0.5*	7.0 ± 0.8*
	Women	1.8 ± 0.1	2.9 ± 0.5	4.3 ± 0.5
Power output (W kg^-1^)^LUM^	Men	3.0 ± 0.2*	4.9 ± 0.6*	7.8 ± 0.9
	Women	2.2 ± 0.2	3.6 ± 0.7	5.2 ± 0.7*
Cycle time (s)	Men	1.52 ± 0.19	1.09 ± 0.11	0.77 ± 0.09
	Women	1.45 ± 0.12	1.05 ± 0.10	0.84 ± 0.10
Poling phase (% of cycle time)	Men	43 ± 4	46 ± 4*	57 ± 2
	Women	47 ± 5	53 ± 3	57 ± 2
Trunk angle at start of poling (°)	Men	71 ± 3	67 ± 8	57 ± 3
	Women	71 ± 5	64 ± 8	58 ± 9
Trunk angle at end of poling (°)	Men	40 ± 8*	35 ± 17	26 ± 10
	Women	51 ± 6	41 ± 10	34 ± 9
Range of motion of the trunk (°)^a^	Men	36 ± 9*	39 ± 13	39 ± 11
	Women	25 ± 7	30 ± 8	33 ± 9
Range of motion of the shoulder (°)^a^	Men	101 ± 13	105 ± 19	99 ± 20
	Women	101 ± 19	102 ± 18	91 ± 27
Range of motion of elbow flexion (°)^a^	Men	36 ± 15	38 ± 21	37 ± 24
	Women	27 ± 22	31 ± 23	22 ± 18
Range of motion of elbow extension (°)^a^	Men	65 ± 9	71 ± 8	75 ± 11
	Women	70 ± 7	79 ± 11	81 ± 17

*TUM* normalized for total upper-body mass, *LUM* normalized for lean upper-body mass

^a^ From the start until the end of the poling phase

* significantly different from the corresponding value for women

### Kinematics

During the submaximal, 3-min and 30-s poling tests, the men produced 97%, 102% and 91% more work per cycle, respectively (all *P* < 0.001, Fig 2), with no significant difference between these values. The cycle rate for the men and women did not differ significantly during any test, but there was an interaction effect between gender and intensity, with a larger range in the cycle rate among the men (*P* < 0.001).

The poling phase constituted approximately half of the total cycle time and was significantly shorter for the men than the women during the 3-min test (*P* < 0.01, Table 2). The relative duration of the poling phase increased with increasing exercise intensity (*P* < 0.05). The trunk angle at the start of the poling phase did not differ between genders, whereas at the end of the poling phase this angle was smaller and the range of trunk motion was greater for the men at all exercise intensities, although only significantly so at submaximal intensity (*P* < 0.05, Table 2). There were no gender differences in the range of motion of the shoulder and elbow joints during any test. The velocity of trunk flexion during the poling phase was significantly higher for the men in connection with all three tests (all *P* < 0.05, Fig 2), without any interaction effect with intensity. The velocities of shoulder extension and elbow flexion were significantly higher for the men only during the 30-s test (*P* < 0.05, Fig 2), without any differences in the velocity of elbow extension.

### Physiology

Oxygen uptake (in L min^-1^) was 61% higher among the men during both the submaximal and 3-min tests (both *P* < 0.001, Table 3) and reduced to **~**23% and **~**13% when normalized for total and lean upper-body mass, respectively (see body composition values in Table 4) (all *P* < 0.05, Table 3). When expressed as a percentage of running VO_2_max, the men still utilized a significantly higher oxygen uptake (both *P* < 0.01, Table 3). The oxygen pulse (both in absolute terms and relative to the maximal value when running) was significantly higher among the men (all *P* < 0.05), since there were no significant gender differences in heart rate or percentage of HRmax. The blood lactate concentration after the submaximal test did not differ between the men and women, but was significantly higher for the men following the 3-min test (*P* = 0.04, Table 3) and tended to be higher for the men after the 30-s test (*P* = 0.08, Table 3).

**Table 3:** Physiological responses (means ± SD) of the 8 male and 8 female elite cross-country skiers during upper-body poling at submaximal intensity and during a 3-min maximal performance and 30-sec all-out test.

		Intensity		
		Submaximal	3-min	30-s
Oxygen uptake (L min^-1^)	Men	2.7 ± 0.5*	4.3 ± 0.7*	-
	Women	1.7 ± 0.2	2.7 ± 0.3	-
Oxygen uptake (mL min^-1^ kg^-1^)	Men	35.1 ± 3.4*	56.1 ± 4.8*	-
	Women	26.0 ± 2.3	41.7 ± 3.5	-
Oxygen uptake (mL min^-1^ kg^-1^)^TUM^	Men	57.0 ± 5.6*	91.1 ± 7.9*	-
	Women	46.1 ± 4.4	74.2 ± 7.9	-
Oxygen uptake (mL min^-1^ kg^-1^)^LUM^	Men	63.9 ± 6.0*	102.0 ± 8.7*	-
	Women	56.0 ± 5.9	90.1 ± 11.9	-
% of VO_2_max^a^	Men	48 ± 5*	76 ± 6*	-
	Women	42 ± 4	67 ± 6	-
Oxygen pulse (mL beat^-1^)	Men	18.5 ± 3.4*	24.0 ± 4.0*	-
	Women	11.3 ± 1.4	15.3 ± 2.1	-
% of maximal oxygen pulse^a^	Men	64 ± 8*	83 ± 7*	-
	Women	55 ± 4	74 ± 5	-
Heart rate (bpm)	Men	148 ± 13	181 ± 5	176 ± 5
	Women	150 ± 13	178 ± 7	169 ± 10
% of maximal heart rate^a^	Men	75 ± 6	91 ± 2	89 ± 2
	Women	77 ± 5	91 ± 2	86 ± 4
Blood lactate (mmol L^-1^)	Men	4.1 ± 1.5	13.3 ± 2.2*	9.4 ± 2.0
	Women	4.0 ± 1.1	11.1 ± 1.7	7.7 ± 1.5

*TUM* and *LUM* were normalized for total and lean upper-body mass, respectively (see these values in Table 4)

^a^ Reached during treadmill running

* significantly different from the corresponding value for women

**Table 4:** Total body, lean and fat mass of the 8 male and 8 female elite cross-country skiers, presented as absolute and percentage values, and relative to total body mass (%TBM) (means ± SD).

				Mass			
		Total		Lean		Fat	
		kg	%TBM	kg	%	kg	%
Total body	Men	78.4 ± 7.3 *	-	67.7 ± 6.0*	86 ± 2*	8.0 ± 1.7*	10 ± 2*
	Women	65.2 ± 5.9	-	50.6 ± 4.2	78 ± 2	12.3 ± 2.4	19 ± 3
Upper body	Men	47.6 ± 5.1*	61 ± 2*	42.4 ± 4.4*	89 ± 1*	3.9 ± 0.9*	8 ± 2*
	Women	36.5 ± 3.6	56 ± 3	30.1 ± 2.8	83 ± 3	5.5 ± 1.4	15 ± 3

* Significantly different from the corresponding value for women

### Body composition

The total body, lean and fat mass of the men was 20% and 34% higher and 35% lower, respectively, than for the women (all *P* < 0.001, Table 4), with corresponding values of 30%, 41% and 29% for the upper body (all *P* < 0.001, Table 4). A significantly greater portion of the total body mass was localized in the upper body of the men (61% vs 56%, *P* = 0.002, Table 4).

### Training data

There were no significant gender differences in the total number of training hours or in the extent of endurance and speed training during the six months prior to this study. The men performed more than twice as much strength training than the women did (*P* = 0.04, Table 5) and 50% more roller skiing using the classical technique; whereas the women ran significantly more (both *P* < 0.01, Table 5). The questionnaires and interviews indicated that on average ~35%, ~55% and ~10% of the strength training of both men and women was focused on the arms, core and legs, respectively. The ratio between maximal strength and submaximal strength training was reported to be approximately 50/50 for the men and 25/75 for the women. When roller skiing, the men reported to choose the double-poling and kick double-poling classical techniques more frequently, whereas most of the women reported to prefer the diagonal stride sub-technique. When skating, both the men and women reported using predominantly the G3 (i.e., V2) sub-technique, but the men utilized this technique relatively more than the women, who employed the G2 technique (i.e., V1) relatively more often.

**Table 5:** Characteristics of the training during the six months prior to testing (May-October) as reported by the 8 male and 8 female elite cross-country skiers.

	Men	Women
Endurance training		
Low-intensity (h)	278 ± 45	278 ± 40
Moderate-intensity (h)	15 ± 4	13 ± 4
High-intensity (h)	18 ± 7	19 ± 5
Speed training (h)	10 ± 5	10 ± 5
Strength training (h)	43 ± 25*	20 ± 11
**Total hours**	**364 ± 60**	**337 ± 49**
Classical roller-skiing (h)	97 ± 13*	64 ± 11
Skate roller-skiing (h)	68 ± 11	63 ± 11
Running (h)	104 ± 14	158 ± 41
Other training modes (h)	40 ± 49	26 ± 7

* Significantly different from the corresponding value for women

## Discussion

The current study examined the impact of exercise intensity on gender differences in the performance of upper-body ergometer poling among cross-country skiers. Our primary finding was that the gender difference in power production was augmented by elevating the intensity of exercise. Specifically, this difference rose from 88% during low-intensity submaximal poling to 95% during the 3-min test and 108% during the 30-s test. The gender difference in the peak power pull-down test was even greater, i.e., 118%. These relative gender differences are more pronounced than those previously observed in connection with lower- and whole-body exercise, and coincide with higher peak aerobic capacity, greater upper-body maximal strength, more muscle mass in the upper-body, and more extensive upper-body strength and endurance training by the male skiers.

While the main difference between genders in ergometer poling reflected differences in the work performed per cycle, the cycle rate of the men was also more elevated at higher exercise intensities. At the same time, men exhibited more rapid trunk movements during all tests and had faster shoulder extension and elbow flexion at the highest intensity. Additionally, the men demonstrated a 61% higher peak oxygen uptake during poling and a 58% higher 1RM in the pull-down test, had relatively more upper-body mass and performed significantly more upper-body strength and endurance training.

The enhanced gender difference in power output with increasing intensity of ergometer poling, as well as the even greater difference in power in connection with the peak power pull-down test observed here contrasts with previous reports that intensity does not influence gender differences during whole- and lower-body exercise [4, 5]. In a practical context, the influence of exercise intensity observed here corresponds to larger gender differences during competitions of shorter duration. In addition, the gender differences in power output revealed in our study are higher than those reported previously for whole- and lower-body exercise [1, 4, 23, 24]. Accordingly, we conclude that gender differences are greater for upper- than lower-body exercise and that these differences become more pronounced when higher power is required.

The gender difference in power production during ergometer poling documented here reflected primarily differences in the work performed per cycle, a measure comparable to cycle length, which is a typical determinant of performance during cross-country skiing [25, 26]. Furthermore, the increase in cycle rate from submaximal poling to the 30-s test was more pronounced for the men, who demonstrated higher angular velocities during trunk flexion at all exercise intensities and more rapid extension of the elbow joint and flexion of the shoulder during the 30-s test. These gender differences in technique are typical for the differentiation between faster and slower double-poling performance [27] and indicate that, in addition to the force produced, the speed of movement also contributes to the elevated gender difference in upper-body power at higher intensities. This conclusion is further supported by the observation that the gender difference in 1RM pull-down strength was approximately half of the difference in peak power output involving the same movement.

These extensive gender differences in upper-body power coincided with 61% higher peak oxygen uptake by the men, a greater difference than those reported previously in connection with whole-body skiing techniques [1, 5]. Moreover, during upper-body poling the men came closer to their VO_2_max than the women (76% versus 67%). Similarly, male elite skiers attain 78% of their VO_2_max during arm cranking [28]. However, the present study is the first to compare the physiological responses of elite male and female cross-country skiers during isolated upper-body exercise. Since there was no gender difference in heart rate during any of the tests, the differences in VO_2_ must be solely due to variations in factors influencing the O_2_ pulse. This implies that men exhibit a larger stroke volume, utilize a relatively larger proportion of their maximal stroke volume and/or extract oxygen more effectively during upper-body poling. All these factors are likely related to differences in the upper-body composition and training between men and women, which are aspects that require further elaboration. While these factors may all contribute to explain the large gender differences in the production of upper-body power, future studies are required to elucidate interactions between these variables, as well as their direct effects on power production at different intensities of exercise.

The higher amount of lean mass in the upper body of men indicates that higher muscle mass contributes to their greater output of power, higher 1RM strength and peak aerobic capacity, as well as their greater anaerobic capacity [7, 29]. The latter proposal is supported by the pronounced differences in the 30-s all-out test, a reliable and valid indicator of anaerobic capacity in connection with other sports [30]. Overall, the power differences were reduced by more than 50% when normalized for lean upper-body mass. Nonetheless, 33–48% of these differences remained, in contrast to previous reports that such gender differences are obliterated by this type of normalization [6, 13]. Normalizing VO_2_peak for lean upper-body mass reduced the gender difference from 61 to 13%, with the remaining difference likely being explained by the higher hemoglobin mass of men.

Although such biological differences probably explain a large part of the pronounced gender differences in upper-body poling documented here, the greater magnitude of these differences in comparison to those observed during other types of exercise may reflect variations in training. The complex nature of cross-country skiing requires training a number of different techniques on varied terrain, thereby involving changes in loading of the upper- and lower-body. Here, our data indicate that the men put greater emphasis on upper-body endurance training and, for example, more frequently utilize upper-body dominant techniques such as double poling and G3 skating during their roller ski sessions. Choice of technique in cross-country-skiing is strongly influenced by the terrain and speed [31, 32] and since female skiers attain lower speeds, they typically use the lower-gear techniques where the upper body is less loaded, to a greater extent than male skiers. Our male skiers also trained upper-body strength in the gym more than twice as much and reported an almost four-fold greater emphasis on maximal upper-body strength training. Since maximal upper-body strength training in cross-country skiers is normally performed with similar movement patterns as used in the tests employed here and with high loads, strength training may have especially contributed to the increased gender differences in the 30-sec all-out and the peak power tests. Such differences may accentuate physiological gender differences in the capacities of the upper- and lower-body, an aspect that coaches and athletes should take into consideration when developing training strategies.

The data documented here indicate differences in training between male and female skiers, however, more precise analyses of upper- and lower-body loading and utilization of different skiing techniques during training are required. In this connection, recent advances in sensor technology, that allow monitoring of movement and technique during both training and competitions, will be of considerable value.

### Methodological considerations

Since we employed ergometer double poling to compare the upper-body power production of male and female skiers in an isolated fashion, our present findings cannot be applied directly to performance on snow. Additionally, ergometer poling differs distinctly from double-poling skiing in some ways. On the ergometer, the poling phase generally occupies approximately half of the cycle time, a value higher than during rapid double poling on snow or roller-skiing [27]. Furthermore, the absolute power differences measured while ergometer poling are much greater than the corresponding differences in skiing performance on snow, where taller and heavier skiers work against greater air drag and snow friction. Nonetheless, the patterns of muscle activity associated with ergometer poling and double poling on snow are similar [33] and the former is therefore considered to be a valuable testing procedure.

Recently, Sylta and colleagues [34] concluded that self-reported information concerning the duration and intensity of endurance training of elite athletes is in agreement with their heart rate profiles. Nevertheless, such information, even in combination with our questionnaire, does not provide a complete picture of training among cross-country skiers. For example, in connection with self-reporting ski and roller ski training are divided simply into the classical and skating techniques, but should be divided further into specific sub-techniques. Additionally, strength training is often recorded in terms of hours, rather than by load, sets and numbers of repetitions. These aspects require more attention in the future.

## Conclusion

With the current protocol, gender differences in upper-body power produced by elite cross-country skiers are augmented as the intensity increases. These differences are more pronounced than those observed previously in connection with lower- and whole-body sports and coincide with large differences in delivery of aerobic energy and maximal strength. Additionally, our findings indicate that the upper-body anaerobic capacity and the ability to execute poling rapidly are of considerable importance. Since male skiers have more upper-body muscle mass and train upper-body endurance and strength more extensively, our present observations indicate that female skiers can potentially improve their upper-body capacity considerably. However, this needs to be tested in controlled intervention studies.
